# The effect of ageing on fat infiltration of thigh and paraspinal muscles in men

**DOI:** 10.1007/s40520-022-02149-1

**Published:** 2022-05-28

**Authors:** Klaus Engelke, Mansour Ghasemikaram, Oliver Chaudry, Michael Uder, Armin M. Nagel, Franz Jakob, Wolfgang Kemmler

**Affiliations:** 1 Department of Medicine III, Friedrich-Alexander University of Erlangen-Nürnberg, University Hospital Erlangen, Ulmenweg 18, 91054 Erlangen, Germany; 2 Institute of Medical Physics (IMP), Friedrich-Alexander-Universität Erlangen-Nürnberg (FAU), Henkestr. 91, 91052 Erlangen, Germany; 3 Institute of Radiology, Friedrich-Alexander-Universität Erlangen-Nürnberg and University Hospital Erlangen, Maximiliansplatz 3, 91054 Erlangen, Germany; 4 Bernhard-Heine-Center for Locomotion Research, University of Würzburg, Brettreichstrasse 11, 97074 Würzburg, Germany

**Keywords:** MRI, Thigh, Erector spinae, Psoas, IMAT, Muscle fat fraction

## Abstract

**Background:**

Myosteatosis, skeletal muscle fat infiltration, is associated with inflammation and fibrosis. The age-related increase of myosteatosis is an important characteristic of sarcopenia and contributes to fragility.

**Aims:**

To investigate the impact of healthy aging on intermuscular adipose tissue (IMAT) and muscle fat fraction (FF) in the thigh and the paraspinal muscles in males.

**Methods:**

In 54 healthy males (age 20–70), all active hobby golfers, magnetic resonance imaging was performed to determine volume of IMAT, volume of muscle tissue (MT) and of percentage of FF.

**Results:**

Between ages 20–70, at the thigh, IMAT/MT volume and MT FF increased annually by 2.9% and 1.3%, respectively. At the psoas IMAT/Psoas volume did not change with age. MT FF increased by 1.5% annually. At the erector spinae IMAT/Erector volume decreased by 0.3% and MT FF increased by 2.8% annually.

**Discussion:**

With increasing age, in males, thigh muscle atrophied, muscle tissue was partly replaced by adipose tissue and remaining muscle tissue also contained more fat. Similar effects were observed in the erector spinae. The psoas muscle did not atrophy, although MT FF also increased with age. Overall correlations with age were weak to moderate with higher correlations observed in the paraspinal muscles.

**Conclusions:**

Age-related increases of muscle fat infiltration were observed in the thigh and in the spine. Muscle atrophy did not occur in the psoas. In cross-sectional studies, an adjustment of volumetric parameters by muscle volume is advisable when comparing age-dependent results.

**Supplementary Information:**

The online version contains supplementary material available at 10.1007/s40520-022-02149-1.

## Introduction

According to the latest definition of the European Working Group on Sarcopenia in Older People 2 (EWGSOP2), muscle quantity and quality are part of the sarcopenia case-finding algorithm [1]. A decrease in muscle volume is associated with a decrease of muscle strength and consequently of physical function [2]. However, muscle strength is not just a function of muscle volume. In older adults, the age-related decrease of muscle strength is about three times as high as the decrease of appendicular lean mass measured by dual X-ray absorptiometry (DXA), which is a surrogate of muscle mass [3]. Myosteatosis (skeletal muscle fat infiltration) is another important component of mobility and muscle strength [4–8], which is highly associated with muscle fat fraction [9, 10].

Myosteatosis of the thigh for example, includes the adipose tissue and lipids beneath the fascia lata. For the purpose of this study, two components are differentiated. The first one is IMAT (intermuscular adipose tissue) defined as "visible storage of lipids in adipocytes located between the muscle fibers (also termed intramuscular fat) and also between muscle groups (literally intermuscular)” [4, 11]. In T1-weighted MR (magnetic resonance) images the signal intensity of IMAT appears white whereas the signal intensity of the complement of IMAT, which is termed muscle tissue (MT) appears dark. Traditionally, radiologists assessed muscle fat infiltration by semi quantitatively grading IMAT of the thigh or calf using CT or MR images [12]. Intramuscular fat may be deposited in two distinct compartments, either as intramyocellular lipids (IMCLs) accumulated in the cytoplasm of myocytes or as extramyocellular lipids (EMCLs) in interstitial, intramyofascial adipocytes.

The second component of myosteatosis is not detectable (invisible) in T1-weighted MR images. This component consists of small aggregates of adipocytes between muscle bundles and intramyocellular lipids. However, their combined contribution to myosteatosis can be quantified indirectly from MRI Dixon sequences as fat fraction (FF) of MT [13, 14]. A separation of IMCLs and EMCLs can be achieved by MR spectroscopy but not by MR imaging [15]. An accurate separation of IMAT from muscle tissue of the thigh or calf requires a segmentation of the deep fascia lata (FL) [16], a highly complex network of extracellular matrix and cells of mesenchymal and neural origin [17].

Compared to young healthy subjects, in sarcopenic subjects IMAT volume of lower extremity tissues is increased [18] and in older adults IMAT is associated with decreased mobility [19]. A small Japanese study also reported that between 20 and 70 years the increase of intramuscular adipose tissue volume normalized to muscle volume was larger than the decrease of muscle cross-sectional area normalized to body weight [20]. IMAT was also higher in frail versus non-frail individuals and this was significantly associated with inflammation as analyzed by muscular IL-6 expression [21].

We demonstrated a larger effect of a high intensity training on IMAT than on muscle tissue fat fraction [22] Bruseghini et al. also reported a decrease of IMAT volume after high intensity training in a small group of elderly men [23]. Recently Farrow et al. [24], Hogrel et al. [25] and Yoon et al. [26] using MRI and Delmonico et al. [27] using CT showed age-related increases of fat fraction of the complete thigh muscle ensemble [25–27] and also of individual thigh muscles [24]. However, none of the three studies performed a FL segmentation and IMAT volume was not determined. Also differences between male and female subjects were not presented. An age-related increase of IMAT of the quadriceps muscle of elderly inpatients (> 65 years) was also reported using ultrasound [28]. In abdominal CT images of Chinese women, Peng et al. [29] showed an age-related increase of IMAT and a decrease of muscle cross-sectional area of the paraspinal muscles. An age-related increase of paraspinal FF has also been detected by MRI [30].

To better understand mechanisms of myosteatosis, it is important to separate disease-related effects from those of healthy aging and to investigate the impact of healthy aging on the different components of myosteatosis. Age-related changes must be determined separately for males and females and for different ethnicities—a long to do list. In this study, we explored age-related changes of IMAT volume and muscle tissue fat fraction of the thigh and the paraspinal muscles in active healthy white males.

## Methods

In this study, baseline data of the Franconian EMS and Golf (FREMGO) study [31] were used. FREMGO has been approved by the Friedrich-Alexander University Erlangen-Nürnberg (FAU) Ethics Committee (number 377_19b) and was registered under ClinicalTrials.gov: NCT04264416. The study fully complied with the Helsinki Declaration “Ethical Principles for Medical Research Involving Human Subjects” [32]. All study participants gave their written informed consent after having received detailed information.

### Participants

The baseline data of FREMGO consisted of 54 normal healthy German males of age 20–70. All were active hobby or amateur golfers (handicaps: 8–54) of local clubs in Northern Bavaria. None of them carried out athletic sportive activities. Resistance exercise for more than 60 min/week during the 12 months prior to baseline assessments was an exclusion criterion.

### Assessments

Assessments included body composition, MRI of the thigh and paraspinal muscles and a standardized questionnaire for demographic parameters, pain, diseases, injuries and surgery. Participants were requested to refrain from intense physical activity and exercise 48-h pre-testing. Assessments were always performed at the same time of the day (± 90 min), in the same order and by the same researcher using identically calibrated devices.

### Body composition

Body height was assessed by a stadiometer. Body composition and appendicular skeletal muscle mass (ASMM) were measured using a direct-segmental, multi-frequency bio impedance analysis device (DSM-BIA, InBody770, Seoul, Korea).

### Magnetic resonance imaging of the thigh

**Fig. 1:**
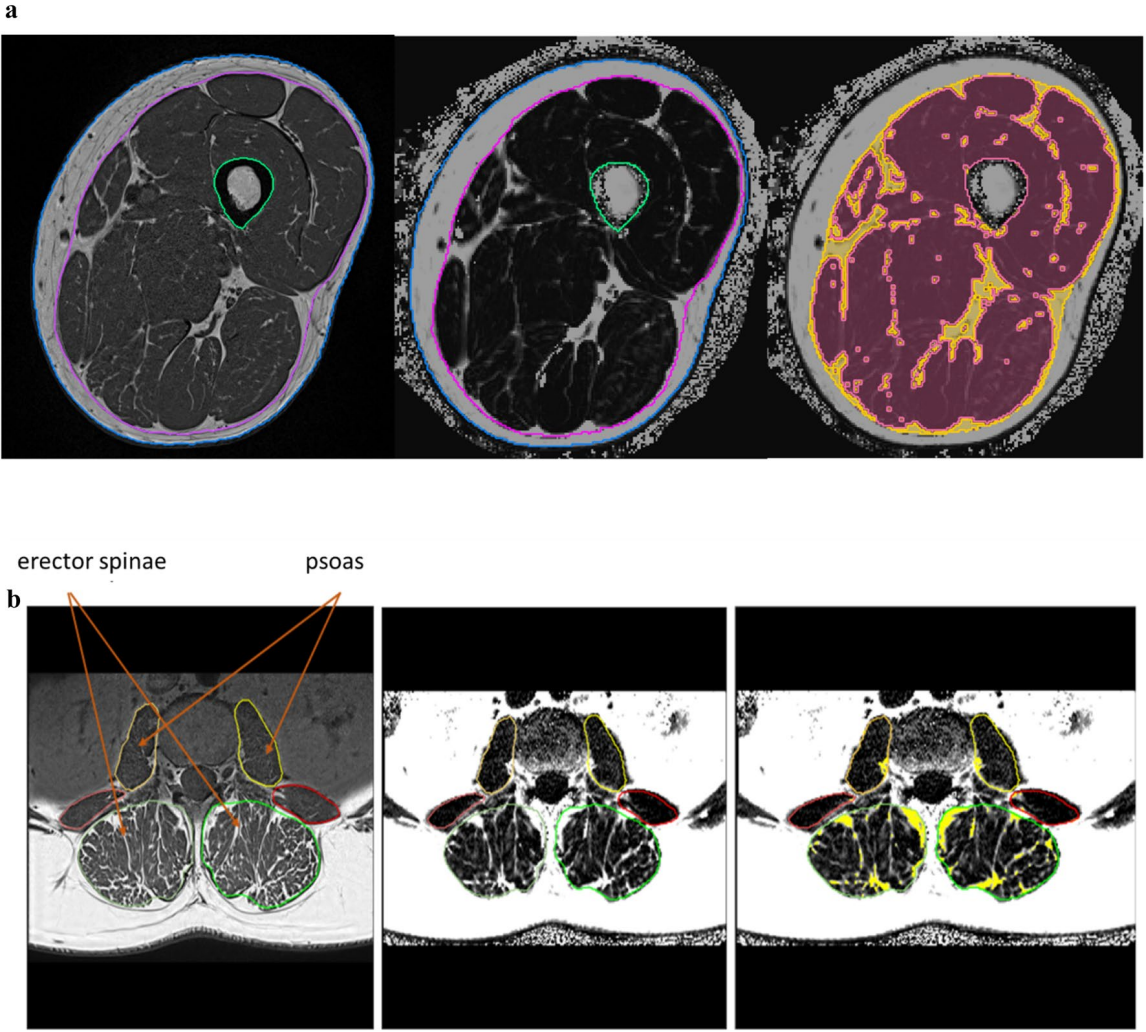
**a** MRI of the thigh. Left: T_1_ weighted sequence with segmented fascia lata; center: Dixon fat fraction image; right: separation of IMAT (yellow) and muscle tissue (red). **b** MRI of paraspinal muscles. Left: T_1_ weighted sequence with segmented muscles; center: Dixon fat fraction image; right: separation of IMAT (yellow) and muscle tissue (dark)

MRI acquisitions were performed on a 3T scanner (MAGNETOM Prisma Fit, Siemens Healthineers AG, Erlangen, Germany) using an 18-channel body surface coil. The protocol included a T1-weighted Turbo Spin Echo and a 6-point Dixon Gradient Echo sequence (Fig. 1a) to determine fat fraction (FF) [14]. For theT1 weighted sequence the following parameters were used: voxel size 0.5 × 0.5 × 3.0 mm^3^, 34 slices, matrix size 512 × 512, TR 844 ms, echo time (TE) 14 ms, bandwidth 488 Hz/px, acquisition time 2:54 min. For the 6pt Dixon sequence the following parameters were used: voxel size 1.0 × 1.0 × 3.0 mm3, 36 slices, matrix size 256 × 256, TR 14.0 ms, TEs 1.90, 3.73, 5.56, 7.39, 9.22 and 11.05 ms, bandwidt 850 Hz/px, flip angle 6°, acquisition time 1:17 min. Intensities of the Dixon FF images ranged from 0 to 1000 corresponding to a FF of 0–100%.

### Magnetic resonance imaging of the spine

At the spine (Fig. 1b) axial slices with a thickness of 3 mm were acquired from mid L1 to mid S1. For theT1 weighted sequence the following parameters were used: voxel size 0.4 × 0.4 × 3.0 mm^3^, 44 slices, matrix size 512 × 336, TR 540 ms, echo time (TE) 11 ms, bandwidth 227 Hz/px, acquisition time 4:57 min. For the 6pt Dixon sequence the following parameters were used: voxel size 0.9 × 0.9 × 3.0 mm^3^, 36 slices, matrix size 288 × 168, TR 17.10 ms, TEs 1.90, 3.71, 7.20, 10.68, 12.49 and 14.30 ms, bandwidth 710 Hz/px, flip angle 4°, acquisition time 1:46 min.

### Image analysis

Image analysis was performed by a trained technician using MIAF (Medical Image Analysis Framework, IMP). The analysis started with the segmentation of the fascia lata in the T1 images (Fig. 1a). Segmentation masks generated in the T1 images were then registered to the corresponding Dixon FF images as described before [16]. Muscle and adipose tissue were automatically separated in the Dixon FF images (Fig. 1a) based on the local minimum of the patient-specific histogram of all voxels within the fascia. The actual threshold used for the separation of muscle and adipose tissue was the FF at 50% below the minimum. This approach is similar to the one used by Rossi et al. [33, 34] who used a threshold of 20% below the minimum in T1 weighted images. Rossi et al. did not obtain Dixon images. Volume of the fascia (intra fascia volume) and of IMAT were measured. In addition, FF of muscle tissue (MT), the complement of IMAT within the FL was determined. Individual muscles were not distinguished. For this analysis technique inter- and intra-operator precision errors (root mean square % coefficients of variation) of lower than 1% for IMAT volume and FF have been reported earlier [16].

The same workflow was used in the spine (Fig. 1b) but only one slice at level L2 was analyzed. Right and left psoas and erector spinae muscles were segmented manually using ImageJ (https://imagej.nih.gov/ij/). The multifidus muscle was included in the erector spinae region of interest. Separate results for IMAT and muscle tissue FF were obtained for the psoas and erector spinae muscles as average values of left and right muscles.

### Statistical procedures

Age-related changes were fitted by polynomial regressions using linear and quadratic models. Annual %change was calculated from slope and intercept and the %CV value was calculated from the standard error of the estimate (SEE) of the linear regression and the corresponding mean *y* value. A *P* value of < 0.05 was considered significant. All data analyses were performed using SPSS (IBM SPSS Statistics, Version 26.0. Armonk, NY: IBM Corp).

## Results

### Age-related changes of the thigh

MRI scans of the thigh were available from 48 and of the spine from 44 of the 54 subjects included in FREMGO. One MRI scan of the thigh and one of the spine were not analyzable because of motion artifacts.

Age-related changes of body mass index (BMI), height and weight of the study population did not differ from corresponding changes of the German normal male population [35] shown in Fig S1.

Tables 1 and 2 show results of linear regressions with age of the thigh and spine, respectively. Annual percent changes relative to age 20 are shown if regressions were significant. Tables S1 and S2 show results of quadratic regressions. In these tables, percent changes from 20 to 40 years relative to age 20 and from 40 to 70 years relative to age 40 are shown if regressions were significant.

**Table 1:** Age-related changes of IMAT, MT and FF in the thigh: linear regression results

Variable	Slope	Intercept	% Change/*y*^a^	SEE	*R*^2^	*P*
FL volume (cm^3^)	− 1.9	1687		228	0.02	ns
IMAT volume (cm^3^)	2.1	67.2	1.9	57	0.24	< 0.001
MT volume (cm^3^)	− 4.0	1621	− 0.3	201	0.09	0.05
IMAT volume/FL volume	0.001	0.03	2.0	0.03	0.39	< 0.001
MT volume/FL volume	− 0.001	0.97	− 0.1	0.03	0.39	< 0.001
IMAT volume/MT volume	0.002	0.03	2.9	0.04	0.38	< 0.001
FL FF (%)	0.13	5.7	1.6	2.8	0.37	< 0.001
MT FF (%)	0.044	2.6	1.3	1.0	0.34	< 0.001
IMAT volume/BMI (cm^5^/kg·10^4^)	0.065	2.9	1.5	1.8	0.24	< 0.001
IMAT volume/ASMM (cm^3^/kg)	0.084	2.0	2.3	1.9	0.32	< 0.001
MT FF/BMI (cm^2^/kg·10^2^)	0.001	0.11	0.8	0.04	0.23	< 0.001
MT FF/ASMM (kg^−1^)	0.002	0.082	1.6	0.04	0.31	< 0.001

*ASMM* appendicular skeletal muscle mass, *BMI*: body mass index, *FF* fat fraction, *FL* fascia lata, *IMAT* intermuscular adipose tissue, *MT* muscle tissue, *P* significance of regression coefficient, *R*^*2*^ square of regression coefficient,* SEE* standard error of the estimate

^a^Changes per year are given in % relative to age 20; value not calculated if regression was not significant

**Table 2:** Age-related changes of IMAT, MT and FF of the paraspinal muscle: linear regression results

Variable	Slope	Intercept	% change/*y*^a^	SEE	*R*^2^	*P*
Psoas						
Volume (cm^3^)	− 0.016	8.9		1.5	0.03	ns
IMAT volume (cm^3^)	0.002	0.41		0.4	0.03	ns
MT volume (cm^3^)	− 0.02	8.5		1.5	0.03	ns
IMAT volume/psoas volume	0.001	0.04		0.05	0.02	ns
MT volume/psoas volume	0.001	0.97		0.05	0.02	ns
IMAT volume/MT volume	0.001	0.04		0.06	0.02	ns
FF (%)	0.07	7.0		4.48	0.05	ns
MT FF (%)	0.065	3.4	1.5	1.33	0.36	< 0.001
Erector						
Volume (cm^3^)	− 0.04	21		0.25	0.05	ns
IMAT volume (cm^3^)	0.059	− 0.71	12.6	0.94	0.48	< 0.001
MT volume	− 0.098	21.6	− 0.5	2.62	0.25	< 0.001
IMAT volume/erector volume	0.003	− 0.045	20.0	0.049	0.51	< 0.001
MT volume/erector volume	− 0.003	1.05	− 0.3	0.049	0.53	< 0.001
IMAT volume/MT volume	0.004	− 0.079	400.0	0.07	0.48	< 0.001
FF (%)	0.30	− 1.38	6.5	4.26	0.53	< 0.001
MT FF (%)	0.096	1.55	2.8	1.56	0.48	< 0.001

Changes per year are given in % relative to age 20

*IMAT* intermuscular adipose tissue, *FF* fat fraction, *P* significance of regression coefficient, *R*^*2*^ square of regression coefficient,* SEE* standard error of the estimate

^a^Changes per year are given in % relative to age 20; value not calculated if regression was not significant

**Fig. 2:**
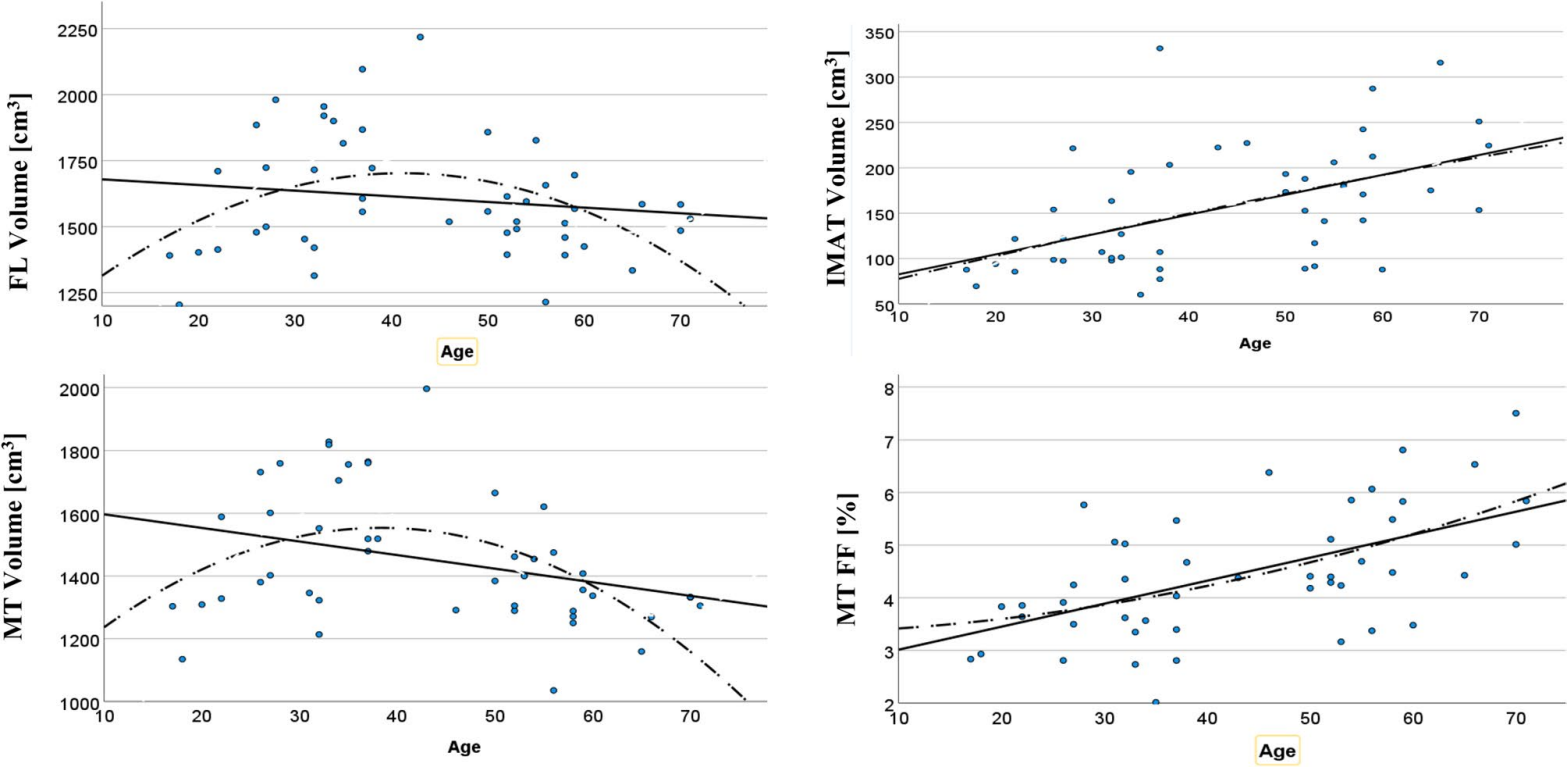
Age-related changes of FL, IMAT and MT volume and MT FF at the thigh. Linear and quadratic fits are shown as solid and dashed lines, respectively

FL and MT volume showed a quadratic dependence of age (Fig. 2) with an increase of 12% for FL volume and 8% for MT volume between age 20 and 40 followed by a decline of 29% for FL volume and 25% for MT volume between age 40 and 70. IMAT volume increased linearly by 1.9% per year. The age-related increase of MT FF could also be approximated by a linear regression with an annual increase of 1.3%.

Correlations of FL, IMAT and MT volume with age were weak (*R*^2^ < 0.25). Normalization of IMAT to MT volume increased the annual percentage changes from 1.9% for IMAT alone to 2.9%. In parallel, the correlation with age increased to *R*^2^ = 0.38. The 1.6% annual increase of FF of the FL was higher than of MT FF (1.3%) because it also reflected the relative increase of IMAT with age. Normalization of IMAT volume or MT FF to BMI or ASMM measured by bio impedance had little effect. If linear regressions were significant, quadratic regressions were not superior in terms of *R*^2^, *P* and SEE.

### Age-related changes of the psoas

**Fig. 3:**
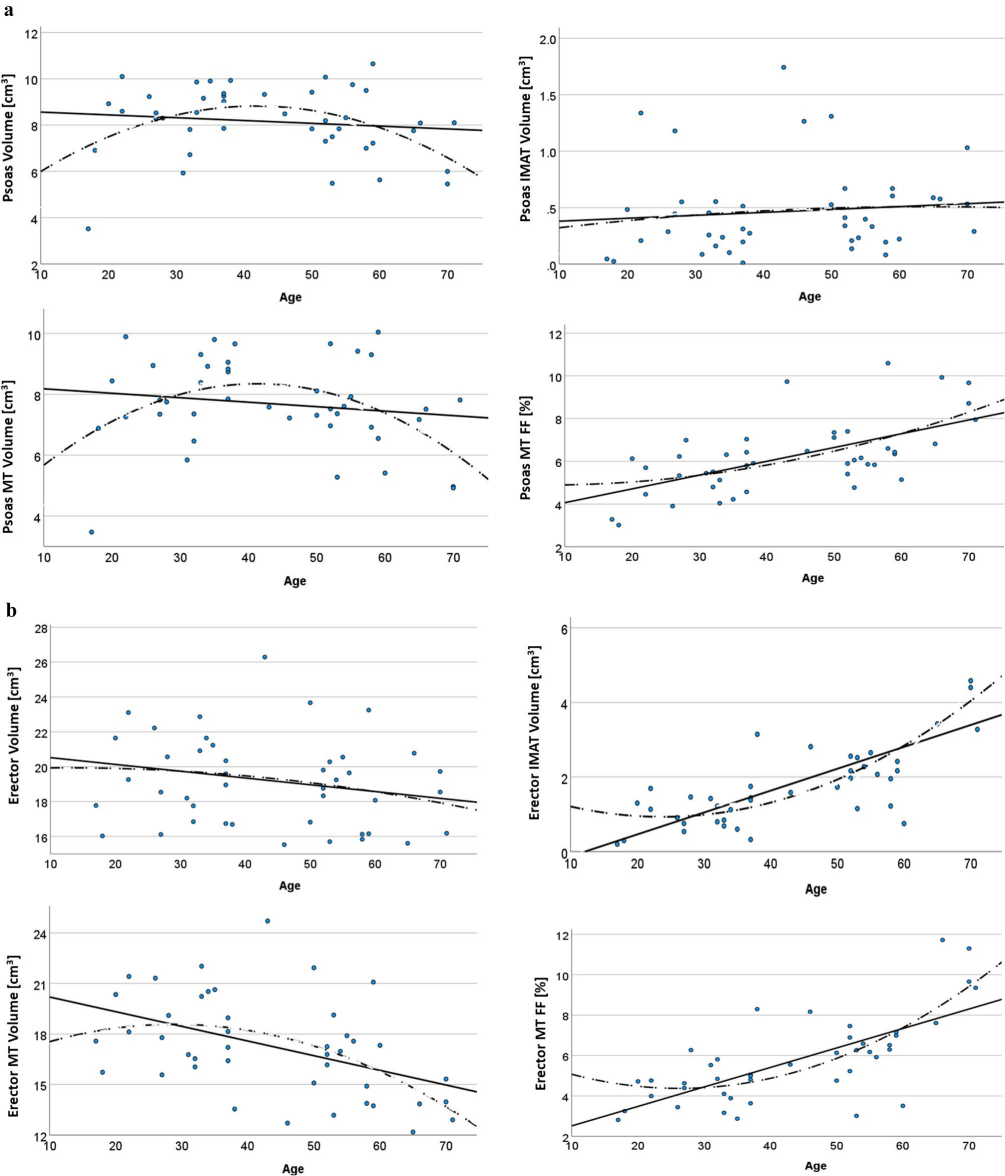
**a** Age-related changes of FL, IMAT and MT volume and MT FF at the psoas muscle. Linear and quadratic fits are shown as solid and dashed lines, respectively. **b** Age-related changes of FL, IMAT and MT volume and MT FF at the erector spinae muscle. Linear and quadratic fits are shown as solid and dashed lines, respectively

Most regressions with age were not significant. Psoas volume and MT volume showed an almost identical quadratic dependence of age (Fig. 3a) with an increase of 13% for FL volume and 14% for MT volume between age 20 and 40 followed by a decline of 35% for FL volume and 37% for MT volume between age 40 and age 70. Consequently, MT/psoas volume and IMAT volume that constituted only 6% of the psoas volume did not significantly change with age. However, there was a significant 1.5% annual increase in MT FF of the psoas.

### Age-related changes of the erector spinae

Erector spinae volume (Fig. 3b) did not significantly decrease with age. In contrast, all other volume and FF measurements showed significant linear increases with age, most notably a 12.6% annual increase of IMAT volume, which resulted in a 20% annual increase of IMAT/erector volume. Linear regressions were highly significant but for the erector, the quadratic regressions resulted in better fits in particular at lower age (Fig. 3b). *R*^2^ values were slightly higher and the fits indicate relative stability of the parameters up to age 40. Thus for the erector annual % changes relative to age 20 shown in Table 2 must be interpreted with caution as the linear fits resulted in very low values at age 20. Consequently, the annual increase of IMAT / MT volume was 400%. The quadratic regression shows an increase of 14% during the first 20 years followed by a 200% increase between age 40 and 70.

## Discussion

In this study, we determined age-related changes of IMAT and muscle tissue fat fraction of the mid-thigh and paraspinal muscles in active healthy males between age 20 and 70. In the thigh, IMAT and muscle tissue fat fraction increased with age but interestingly the dependence of IMAT on age only became significant after adjustment by fascia volume. An increase in IMAT volume combined with a decrease in fascia volume implies a decrease of muscle tissue volume with age. Thus, there was a combination of age-related increase in IMAT volume with concurrent muscle atrophy. In addition, the fat fraction of the remaining muscle tissue further increased. Thus with increasing age, thigh muscle atrophied, muscle tissue was partly replaced by adipose tissue and remaining muscle tissue also contained more fat.

In the spine, similar effects were observed in the erector spinae, in contrast to the psoas muscle, where IMAT volume and muscle tissue volume did not change with age. The psoas muscle as an anatomical structure did not atrophy, however, muscle tissue fat fraction also increased with age. Correlations with age were weak in the thigh and moderate in the paraspinal muscles. Independent of anatomy, correlations of muscle tissue fat fraction with age were higher than for IMAT volume.

Comparison of our results with previously published data is difficult because in many publications the measurement of IMAT and FF is not clearly described. There is also an ongoing discrepancy in the definition of IMAT. We followed the majority of earlier publications [4, 11] defining IMAT as the combination of extracellular adipose tissue between muscle groups and of larger adipocyte agglomerations within muscle, which originated in the early radiological assessment of muscle fat infiltration based on T1-weighted images.

In contrast, a recent Interdisciplinary Workshop at the National Institute on Aging stated that myosteatosis included three components "(a) intermuscular adipose tissue (IMAT), the extra-myocellular adipose tissue found beneath the fascia and in-between muscle groups; (b) intramuscular adipose tissue, the extramyocellular adipose tissue found within an individual muscle; and (c) intramyocellular lipids (IMCL)” [36]. According to this definition, IMAT does not include the extracellular adipose tissue found within an individual muscle. Obviously, standardization of terminology is needed but more importantly, it is still not entirely clear what compartment is most relevant from a medical perspective.

In vivo, IMCL can be assessed with MR spectroscopy [15] but intramuscular adipose tissue cannot be separated from intramyocellular lipids by in vivo imaging due to limited spatial resolution, thus combining larger detectable agglomerations of extracellular adipose with IMAT and separating it from adipose tissue of the remaining muscle tissue seems to be straightforward for in vivo muscle MRI. Also precision errors for MR spectroscopy are much higher than for MRI [13]. The reality is even more complex because in many studies of the thigh the facia lata was not segmented. Instead a tight contour around the muscle ensemble was used. The effect may be small in younger subjects with usually little adipose tissue directly beneath the FL. However, in elderly subjects, without a correct segmentation of the fascia lata IMAT volume will be underestimated regardless of the IMAT definition.

The missing differentiation of the FF of MT in IMCL versus extramyocellular lipids (EMCL), the fat content of interstitial, intramyofascial adipocytes is an important limitation of in vivo MRI. For example, a recent study on back pain using MR spectroscopy showed that EMCL content of the psoas correlated with age, but IMCL content did not [37]. Adipocytes result from differentiation of resident mesenchymal precursors such as Fibro-Adipogenic Precursor (FAP) cells and their expansion. FAP cells can differentiate towards the fibrocyte and the adipocyte pathways [38] and have been shown to be crucial components of muscle regeneration [39]. However, they may also be an origin of fibrotic and fatty infiltration in pathology [40]. ICMLs, lipid droplets (LD) as ubiquitous cellular organelles, provide sources of energy and contribute to the effects of exercise on hypertrophy but also to many other cellular functions like the constitution of the cytoskeletal system and mitochondrial activity [41]. For example, LDs can accumulate in muscle cells in well-trained athletes as a source of energy, but pathologic accumulation of LDs of somewhat different size is associated with insulin resistance, a phenomenon that has been coined ‘the athletes paradox’ [42–44].

Compared to other publications our study has several advantages but also further limitations. We reported data on thigh and paraspinal muscles, but we only included men. The number of less than 50 subjects with available MR images was relatively small but this was also the case for most other studies reporting age-related changes of IMAT or FF. We reported a large variety of combinations of absolute and % values for IMAT and FF. In addition, the age distribution of our cohort was more homogenous than in other studies, in which often just younger and older age groups were compared. However, subjects older than 70 years were not available in our cohort and in particular, the quadratic fits may be inaccurate for age > 65.

For example, Buford et al. [18] showed a 30% increase in IMAT volume of the thigh between elderly (76.4 ± 5.7 years) and younger (23.8 ± 3.9 years) male and female subjects whereas our results in males showed a 60% increase. Part of the discrepancy can probably be attributed to the segmentation of the fascia lata, but exact details were not reported. Farrow et al. [24] compared fat fraction of two thigh muscles among three age groups (26 ± 8 years, 49 ± 19 years, and 79 ± 5 years) of mixed gender. FF of the hamstrings increased with age from 3.4 to 5.6 and 9.5%, i.e., by a factor of 1.6 of the middle aged versus the younger and by a factor of 2.7 of the old versus the young group with similar relative increases in the quadriceps. Our results for MT FF of the whole thigh showed a smaller increase from 3.7% at age 25 to 4.8% at age 50 and 6.1% at age 80 but as discussed above our FF analysis excluded larger agglomerations of intramuscular adipose tissue.

Farrow’s FF analysis was based on segmentation of individual muscles. Typically, this is performed manually and restricted to single slices of the MRI dataset, whereas for the thigh we applied a full 3D analysis and determined FF not in an entire muscle but in muscle tissue. Differences will be small in younger subjects with little intramuscular adipose tissue (Fig. 1a) but in elderly people FF of the complete muscle as determined by Farrow et al. is expected to be higher than FF of the muscle tissue.

Hogrel et al. [25] also performed a segmentation of individual muscles similar to the study from Farrow et al. and compared muscle volume and FF between young (20–30 years) and older (70–80 years) males and females. Similar to our results they also reported an age-related muscle atrophy and a concurrent increase in muscle FF. Decrease in total thigh muscle volume was 22% in men and 21% in women and increase of total thigh muscle FF was 87% in men (compared to 60% for muscle tissue FF in our study) and 68% in women. As a disadvantage, neither Farrow et al. nor Hogel et al. reported IMAT results.

A more comprehensive study was performed by Yoon et al. [26], who measured FF for anterior, medial and posterior thigh muscle compartments. Between 30 and 70 years FF of the posterior compartment increased from 4.4 to 9.8% between youngest and oldest decades compared to an increase of 1.8–2.8% of the anterior and of 1.8–3.9% of the medial compartments. Our data of muscle tissue FF showed an increase from 4.1% at age 35 to 5.5% at age 65.

For the analysis of paraspinal muscles, we performed a manual segmentation of a single slice, only. Crawford et al. [45] reported FF and volume of the erector spinae for 20–60-year-old women and men. In agreement with our results there was no significant age-related change in erector volume and a significant increase in FF. Similar to our results, Dallaway et al. reported a decrease in muscle volume of the erector spinae but not of the psoas in older compared to young males. However, fat infiltration decreased in both muscles [46]. CT data from a large study in 516 Chinese women showed a quadratic increase of IMAT area and of % IMAT from age 20 to age 80 [29]. Of course, it is difficult to compare CT and MRI imaging results but our results showed very comparable age-related changes of IMAT and muscle volume.

From a clinical perspective our data represent an important contribution of imaging to identify and characterize both reversible and irreversible pathologic changes in muscle tissue that could contribute to tissue degeneration and widespread age-associated diseases such as sarcopenia [8, 47], osteoporosis and fragility fractures [48–50] and low back pain [51, 52]. Such findings could both be diagnostic and prognostic markers that influence prevention and treatment decisions. Myosteatosis has recently been recognized as an indicator that reflects metabolic, inflammatory and degenerative pathology and the crosstalk between them [53]. Although myosteatosis is discussed as a separate entity by several authors, it can propagate dysfunction of muscle, bone and tendons, just to name some relevant musculoskeletal tissues. Fatty infiltration of muscle tissue can be initiated by metabolic pathology such as obesity, insulin resistance and diabetes combined with disuse. Alternatively a chronic inflammatory microenvironment may trigger adipogenic and fibrogenic differentiation of muscle stem cells and may further propagate inflammatory myosteatosis. The dystopic and dysregulated adipose tissue secretes pro inflammatory adipokines and as such is part of a vicious cycle that further propagates “inflammaging” and tissue degeneration. Moreover, this microenvironment may also trigger fibrotic scarring as a result of altered tissue regeneration. Taken together the resulting cascades of degeneration promote.

In conclusion and in agreement with other publications in healthy men, thigh muscle volume decreased with age and fat fraction increased. In the spine, similar changes were observed for the erector spinae but not for the psoas. An adjustment of volumetric parameters by FL or MT volume is advisable when comparing age-dependent results in cross-sectional studies as adjusted parameters are more sensitive to muscle atrophy of the thigh. Advanced imaging methods to differentiate EMCL and IMCL effects should be developed.

## Supplementary Information

Below is the link to the electronic supplementary material.

Supplementary file1 (DOCX 57 kb)
