# Supplementary material for: The effect of ageing on fat infiltration of thigh and paraspinal muscles in men
Source: Aging Clin Exp Res. 2022 May 28;34(9):2089–98. doi: 10.1007/s40520-022-02149-1 (PMC9464152; doi:10.1007/s40520-022-02149-1)
Supplement: Supplementary file 1 — Supplementary file1 (DOCX 57 kb) [file 40520_2022_2149_MOESM1_ESM.docx]

1. Supplement

Figure S1: German normal male population: age related changes of BMI. Orange: BMI of study population


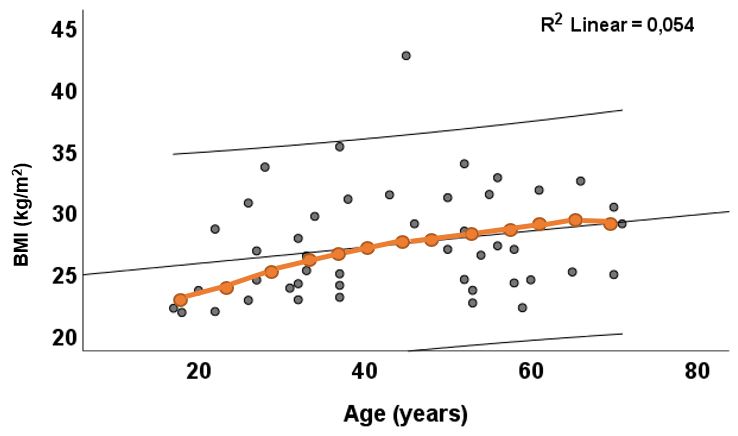


**Table S1.** Age related changes of IMAT, MT and FF in the thigh: Quadratic regressions

| **Variable** | **a** | **b** | **c** | **%change  20 – 40 y** | **%change  40 – 70 y** | **SEE** | **R^2^** | **P** |
| --- | --- | --- | --- | --- | --- | --- | --- | --- |
| FL Volume (cm^3^) | -0.39 | 32.4 | 1030 | 11.8 | -18.5 | 214 | 0.16 | 0.029 |
| IMAT Volume (cm^3^) | -0.011 | 3.0 | 49 | 44.7 | 35.5 | 58 | 0.24 | 0.003 |
| MT Volume (cm^3^) | -0.38 | 29 | 980 | 8.8 | -25.1 | 185 | 0.24 | 0.003 |
|  |  |  |  |  |  |  |  |  |
| IMAT Volume / FL Volume | 1.5 10^-5^ | 0.0001 | 0.59 | 3.3 | 8.5 | 0.03 | 0.40 | <0.001 |
| MT Volume / FL Volume | 1.5 10^-5^ | 0.0001 | 0.94 | 2.1 | 5.4 | 0.03 | 0.40 | <0.001 |
| IMAT Volume / MT Volume | 2.2 10^-5^ | -5.9 10^-5^ | 0.07 | 32.5 | 68.9 | 0.04 | 0.39 | <0.001 |
|  |  |  |  |  |  |  |  |  |
| FL FF | 0.01 | 0.016 | 7.9 | 100.8 | 136.4 | 2.8 | 0.37 | <0.001 |
| MT FF (%) | 0.0005 | 0.005 | 3.3 | 19.4 | 41.9 | 1.0 | 0.34 | <0.001 |
|  |  |  |  |  |  |  |  |  |
| IMAT Volume / BMI (cm^5^/ kg·10^4^) | -0.002 | 0.085 | 2.5 | -20.6 | -150.0 | 1.8 | 0.24 | 0.003 |
| IMAT Volume / ASMM (cm^3^/kg) | -0.0002 | 0.066 | 2.4 | 29.7 | 28.0 | 2.0 | 0.32 | <0.001 |
| MT FF / BMI (cm^2^ kg^-1^·10^2^) | 2.4 10^-5^ | -0.001 | 0.154 | 6.1 | 32.3 | 0.04 | 0.25 | 0.003 |
| MT FF / ASMM (kg^-1^) | 2.8 10^-5^ | 0.001 | 0.13 | 33.3 | 57.0 | 0.04 | 0.33 | <0.001 |

FF: fat fraction; FL: facia lata; IMAT: intermuscular adipose tissue; MT: muscle tissue; R^2^: square of regression coefficient; F: P: p value; SEE: standard error of the estimate. a, b, and c: constants of quadratic regression: y=a x^2^ + b x + c. % change 20 -40y: change in % between age 20 and 40 relative to age 20; % change 40 -70y: change in % between age 40 and 70 relative to age 70.

**Table S2.** Age related changes of IMAT, MT and FF of the paraspinal muscle: quadratic regression results

| **Variable** | **a** | **b** | **c** | **%change  20–40 y** | **%change  40–70 y** | **SEE** | **R^2^** | **P** |
| --- | --- | --- | --- | --- | --- | --- | --- | --- |
| **Psoas** |  |  |  |  |  |  |  |  |
| Volume (cm^3^) | -0.003 | 0.23 | 4.1 | 13.3 | -35.3 | 1.4 | 0.17 | 0.02 |
| IMAT Volume (cm^3^) | 2.7 10^-5^ | 0.001 | 0.5 |  |  | 0.4 | 0.004 | ns |
| MT Volume (cm^3^) | -0.003 | 0.23 | 3.7 | 14.1 | -37.0 | 1.4 | 0.19 | 0.02 |
|  |  |  |  |  |  |  |  |  |
| IMAT Vol / Psoas Vol | 2.8 10^-5^ | -0.002 | 0.087 |  |  | 0.05 | 0.04 | ns |
| MT Vol / Psoas Vol | 2.8 10^-5^ | 0.002 | 0.91 |  |  | 0.05 | 0.04 | ns |
| IMAT Vol/ MT Vol | 3.3 10^-5^ | -0.002 | 0.1 |  |  | 0.06 | 0.04 | ns |
|  |  |  |  |  |  |  |  |  |
| FF (%) | 0.003 | -0.17 | 11.7 |  |  | 4.5 | 0.07 | ns |
| MT FF (%) | 0.001 | -0.012 | 4.9 | 19.0 | 48.8 | 1.3 | 0.37 | <0.001 |
|  |  |  |  |  |  |  |  |  |
| **Erector** |  |  |  |  |  |  |  |  |
| Volume (cm^3^) | -0.001 | 0.015 | 19.8 |  |  | 2.6 | 0.06 | ns |
| IMAT Volume (cm^3^) | 0.002 | -0.087 | 2.2 | 52.4 | 207.8 | 0.9 | 0.55 | <0.001 |
| MT Volume | -0.002 | 0.10 | 17.7 | -2.1 | -19.5 | 2.6 | 0.3 | 0.002 |
|  |  |  |  |  |  |  |  |  |
| IMAT Vol/ Erector Vol | 8.6 10^-5^ | -0.004 | 0.11 | 36.0 | 187.0 | 0.046 | 0.58 | <0.001 |
| MT Vol / Erector Vol | -8.6 10^-5^ | 0.004 | 0.90 | -2.5 | -17.8 | 0.046 | 0.58 | <0.001 |
| IMAT Volume / MT Vol | 0.00014 | -0.008 | 0.162 | 13.8 | 336.4 | 0.067 | 0.56 | <0.001 |
|  |  |  |  |  |  |  |  |  |
| FF (%) | 0.007 | -0.34 | 11.1 | 22.5 | 148.3 | 4.0 | 0.60 | <0.001 |
| MT FF (%) | 0.003 | -0.14 | 6.2 | 17.4 | 105.6 | 1.5 | 0.54 | <0.001 |

FF: fat fraction; FL: facia lata; IMAT: intermuscular adipose tissue; MT: muscle tissue; R^2^: square of regression coefficient; P: p value; SEE: standard error of the estimate. a, b, and c: constants of quadratic regression: y=a x^2^ + b x + c. % change 20 -40y: change in % between age 20 and 40 relative to age 20; % change 40 -70y: change in % between age 40 and 70 relative to age 70.
